# Successful afatinib rechallenge in a patient with non‐small cell lung cancer harboring *EGFR* G719C and S768I mutations

**DOI:** 10.1111/1759-7714.13532

**Published:** 2020-06-11

**Authors:** Tomomi Masuda, Noriaki Sunaga, Norimitsu Kasahara, Kazutaka Takehara, Masakiyo Yatomi, Kenichiro Hara, Yasuhiko Koga, Toshitaka Maeno, Takeshi Hisada

**Affiliations:** ^1^ Department of Respiratory Medicine Gunma University Graduate School of Medicine Maebashi Japan; ^2^ Innovative Medical Research Center Gunma University Hospital Maebashi Japan; ^3^ Gunma University Graduate School of Health Sciences Maebashi Japan

**Keywords:** Afatinib, non‐small cell lung cancer, rechallenge, uncommon *EGFR* mutation

## Abstract

Recent studies have indicated that afatinib is beneficial for patients with non‐small cell lung cancer (NSCLC) harboring uncommon *epidermal growth factor receptor* (*EGFR*) mutations, while the effectiveness of afatinib rechallenge has not been fully defined. Here, we report a long‐term survival case of NSCLC harboring concomitant *EGFR* G719C and S768I mutations who received afatinib rechallenge followed by chemotherapy. The present case suggests that combined therapeutic strategies such as afatinib plus sequential chemotherapy would be beneficial based on appropriately timed rebiopsies from recurrent lesions.

**Key points:**

**Significant findings of the study and what this study adds:**

A NSCLC patient carrying *EGFR* G719X/S768I mutations survived for a long period of time with afatinib rechallenge followed by chemotherapy. Combined therapeutic strategies should be considered based on rebiopsies in appropriate timing in NSCLC with uncommon *EGFR* mutations.

## Introduction

Mutations in the *epidermal growth factor receptor* (*EGFR*) gene is a common driver oncogene in non‐small cell lung cancer (NSCLC). Exon 19 deletions and the L858R point mutation are common *EGFR* mutations within the exon 18–21 tyrosine kinase domain and these mutations are associated with EGFR‐tyrosine kinase inhibitor (EGFR‐TKI) sensitivity and prolonged survival in NSCLC.^1^ Meanwhile, therapeutic strategies for patients with uncommon *EGFR* mutations such as G719X and S768I point mutations have not been well defined.^2^ Here, we report a case of NSCLC harboring *EGFR* G719C/S768I mutations who received afatinib rechallenge followed by chemotherapy.

## Case report

**Figure 1:**
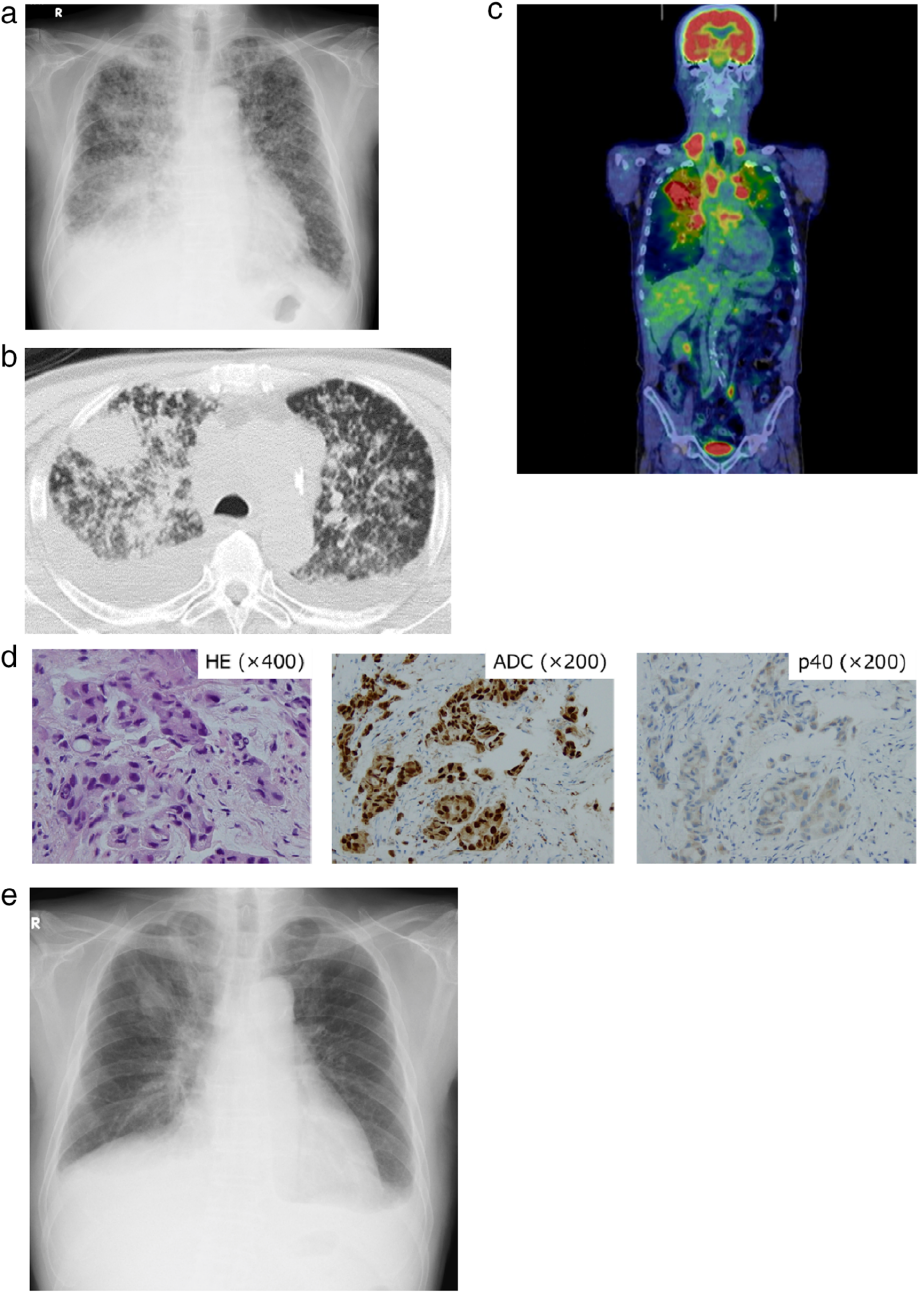
(**a**) Chest X‐ray, (**b**) computed tomography (CT) and (**c**) ^18^F‐fluorodeoxyglucose positron emission tomography (FDG‐PET)/CT represent a mass lesion in the right upper lobe along with mediastinal lymphadenopathy, multiple pulmonary nodules and bilateral pleural effusion before initiating afatinib. (**d**) Histological examination of biopsy specimens from the primary tumor showing adenocarcinoma morphology positive for ADC cocktail antibody staining (TTF‐1 and Napsin A) but negative for p40 antibody staining. HE, hematoxylin‐eosin staining. (**e**) The primary tumor and multiple pulmonary metastases shrank after one month of afatinib initiation.

**Figure 2:**
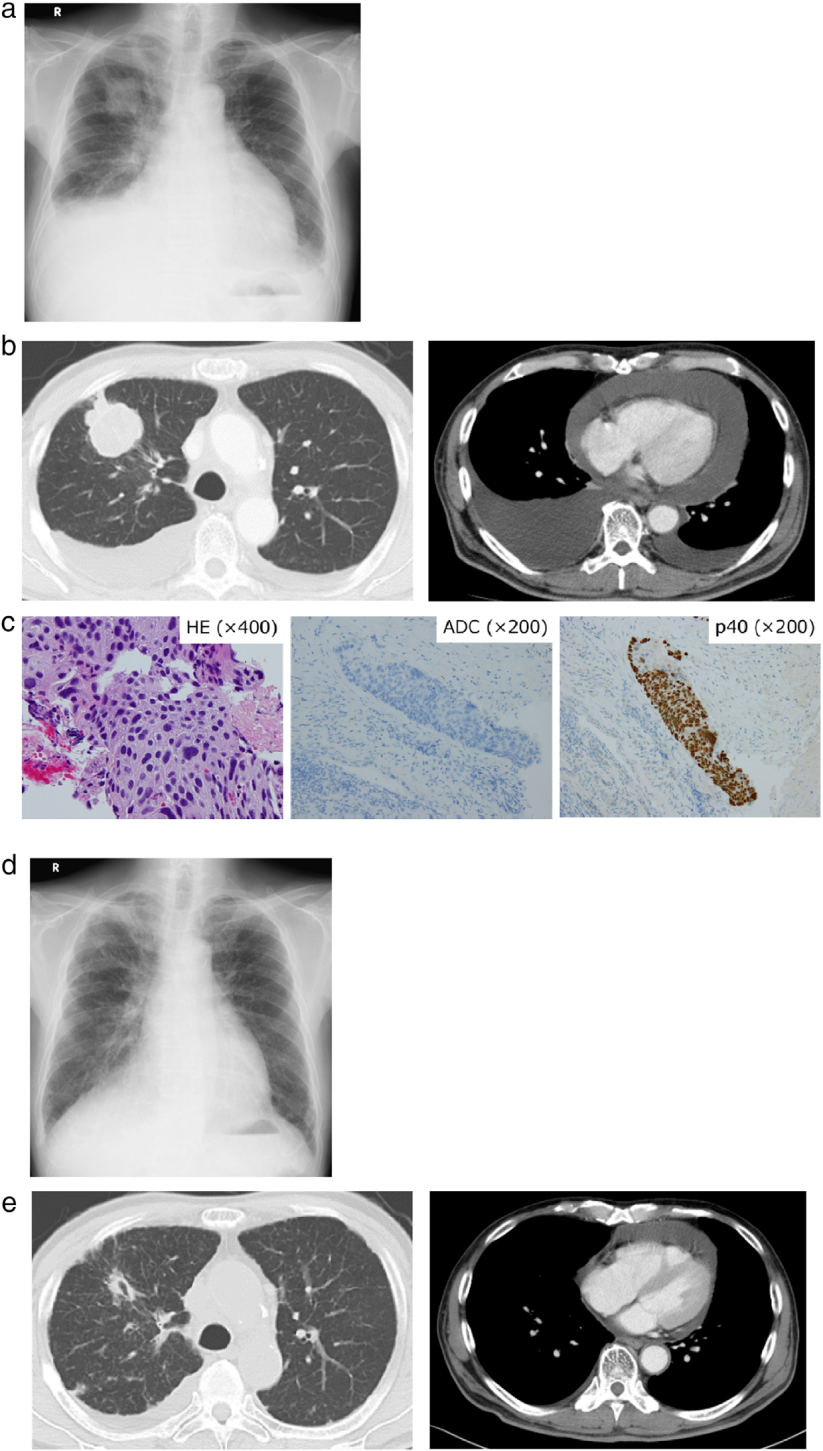
(**a**) Chest X‐ray and (**b**) CT showing regrowth of the primary tumor with increased pleural and pericardial effusions after three months of afatinib initiation. (**c**) Histological examination of rebiopsy specimens from the primary tumor showing squamous cell carcinoma morphology negative for ADC cocktail antibody staining but positive for p40 antibody staining. (**d**) Chest X‐ray and (**e**) CT showing marked regression of the primary tumor with decreased pleural and pericardial effusions after the treatment with CBDCA plus nab‐PTX chemotherapy.

**Figure 3:**
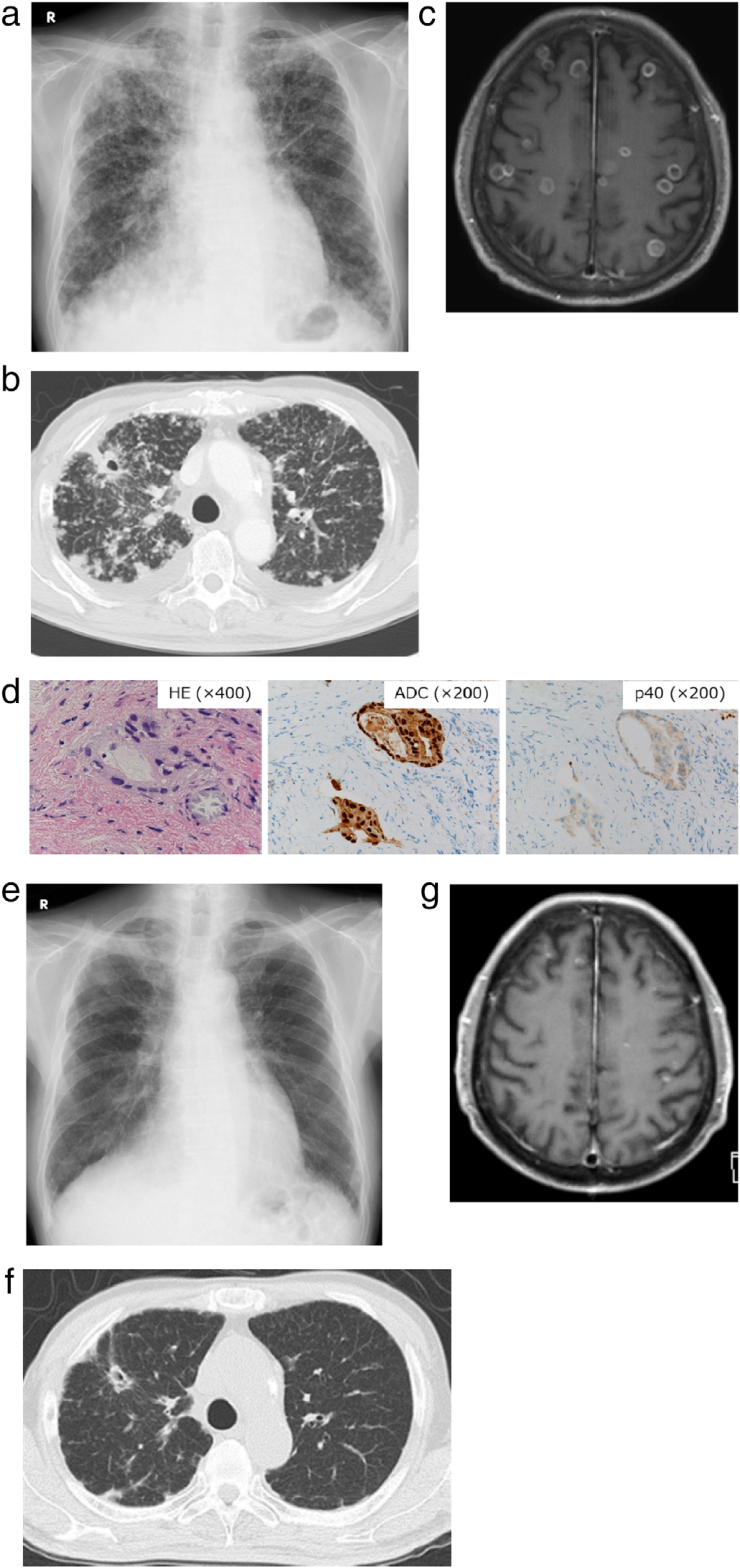
(**a**) Chest X‐ray and (**b**) CT showing apparently‐increasing multiple nodules in the bilateral lung. (**c**) Gadolinium‐enhanced magnetic resonance imaging (MRI) showing multiple lesions with ringed enhancement in the brain. (**d**) Histological examination of rebiopsy specimens from the pulmonary metastatic lesion in the right lower lobe showing adenocarcinoma morphology positive for ADC cocktail antibody staining but negative for p40 antibody staining. Multiple pulmonary and brain metastases regressed after afatinib readministration as shown in (**e**) chest X‐ray, (**f**) chest CT; and (**g**) brain MRI scan.

**Figure 4:**
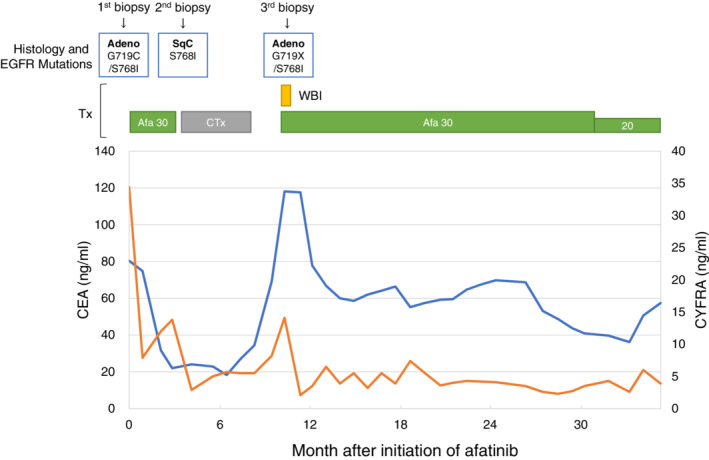
Clinical course with changes of the CEA and CYFRA tumor markers and timing of biopsy from the tumors. As for *EGFR* mutation testing, the MINtS and the PNA‐LNA PCR clamp methods were used for the first biopsy specimen, and the cobas *EGFR* Mutation Test v2 assay was used for the second and third biopsy specimens. Adeno, adenocarcinoma; Afa, afatinib; CTx, CBDCA plus nab‐PTX chemotherapy; SqC, squamous cell carcinoma; Tx, treatment; WBI, whole‐brain irradiation (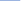 ) CEA, (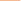 ) CYFRA

A 77‐year‐old man with a 114‐pack‐year smoking history came to the hospital with symptoms of cough, dyspnea and right chest pain in February 2017. Examination of chest X‐ray, computed tomography (CT) and ^18^F‐fluorodeoxyglucose positron emission tomography (FDG‐PET)/CT revealed a mass lesion in the right upper lobe, along with pleural effusion, multiple nodules in the bilateral lung fields, bones and left adrenal gland (Fig 1a–c). Transbronchial lung biopsy from the primary tumor and following *EGFR* mutation testing resulted in a diagnosis of adenocarcinoma harboring concomitant G719C and S768I mutations with no other detectable *EGFR* mutations in exons 18–21 (Fig 1d). Based on a diagnosis as stage IVB lung adenocarcinoma (cT4N3M1c), afatinib at 30 mg once daily was administered starting in March 2017. After one month, the primary tumor and multiple pulmonary metastases had markedly regressed (Fig 1e). However, chest X‐ray and CT showed regrowth of the primary tumor with increased pleural and pericardial effusion after three months of afatinib initiation (Fig 2a‐b). Transbronchial rebiopsy from the primary tumor was performed and histopathological examination revealed that the recurrent tumor was composed of squamous cell carcinoma cells with the S768I mutation, while the G719C mutation was undetectable (Fig 2c). The patient then received CBDCA plus nab‐PTX chemotherapy for six cycles and the primary tumor markedly shrank accompanied by reduced pleural and pericardial effusions (Fig 2d‐e). However, six months after initiation of chemotherapy, multiple pulmonary and brain metastases appeared without regrowth of the primary tumor (Fig 3a–c). Transbronchial lung rebiopsy from a pulmonary metastatic lesion in the right lower lobe was performed again. The pulmonary metastases were found to be composed of adenocarcinomas harboring concomitant G719X and S768I mutations (Fig 3d). He received whole‐brain irradiation for multiple brain metastases because he developed cognitive decline and afatinib was resumed at 30 mg once daily. After one month, multiple pulmonary and brain metastases had dramatically regressed (Fig 3e–g). He has been receiving afatinib and is recurrence‐free 38 months from initiation of treatment up to the present time (May 2020). Clinical course with changes of the CEA and CYFRA tumor markers along with timing of biopsy from the tumors and *EGFR* mutation testing are summarized in Fig 4.

## Discussion

Uncommon *EGFR* mutations include G719X mutations (2%–4%) in exon 18 and a S768I mutation (<5%) in exon 20 and clinical outcome for NSCLC patients with these mutations has been previously reported.^2^ In a combined analysis of LUX‐Lung 2, LUX‐Lung 3 and LUX‐Lung, afatinib exhibited superior treatment efficacy compared to chemotherapy in *EGFR*‐mutated NSCLC harboring these uncommon mutations.^3^ A pooled analysis demonstrated that such uncommon mutations are sensitive to afatinib in the first‐line setting.^4^ Additionally, G719X and S768I mutations frequently coexist with other uncommon mutations and NSCLC patients harboring these compound mutations exhibited high response rates and long duration of response with afatinib.^4^ These studies suggest that afatinib is clinically effective for NSCLC patients with G719X and S768I mutations. Our case did not have a long duration of response to first‐line afatinib, but subsequent chemotherapy followed by afatinib rechallenge achieved long‐term survival. It is noteworthy that information on histological examination and *EGFR* mutation testing acquired by second and third rebiopsies were a great assistance in determining the treatment plan in our case.

While several studies have indicated that EGFR‐TKI rechallenge is a potent therapeutic option,^5^, ^6^, ^7^ clinical effectiveness of afatinib rechallenge remains obscure. A previous report described successful afatinib rechallenge after failure of first‐line afatinib in a NSCLC patient with the *EGFR* exon 19 deletion, who was treated with chemotherapy after progressive disease with afatinib.^8^ Thus, afatinib rechallenge may be effective when subsequent chemotherapy is introduced prior to afatinib readministration.

The mechanisms of acquired resistance to EGFR‐TKIs are mainly caused by the *EGFR* T790M mutation in exon 20, whereas histological transformation from adenocarcinoma to small cell carcinoma or to squamous cell carcinoma rarely occurs.^9^, ^10^ In our case, histological transformation from adenocarcinoma to squamous cell carcinoma was observed on second rebiopsy from the primary tumor with short duration of response to afatinib. It is possible that the primary tumor was comprised of a heterogeneous mixture with adenocarcinoma and squamous cell carcinoma components before afatinib initiation. Tanaka *et al*. investigated rebiopsy specimens in 37 NSCLC patients including three uncommon *EGFR* mutants who had progressed after afatinib treatment and found that the T790M mutation was detected in 43% of them, but T790M was undetectable in all uncommon mutants.^11^ Considering that NSCLC tumors with uncommon *EGFR* mutations represent highly heterogenous molecular characteristics with various clinical responses to EGFR‐TKIs,^12^ molecular heterogeneity may confer the resistant mechanisms and combined therapeutic approach such as afatinib plus chemotherapy seems to be effective for this mutant population.

The present case was initially administered afatinib at 30 mg/day and achieved long‐term survival without any serious adverse event (AE). While the standard commencing dose of afatinib is 40 mg/day, a post‐hoc analysis of LUX‐Lung 3 revealed that dose reductions were made in 53.3% of afatinib‐treated patients due to AEs.^13^ Additionally, a phase II study showed that first‐line afatinib at 30 mg/day was highly effective with acceptable toxicity in elderly patients with NSCLC harboring *EGFR* mutations.^14^ Therefore, afatinib at a dose of 30 mg/day might be considered as a treatment option for elderly patients with *EGFR*‐mutated NSCLC in the first‐line setting.

In summary, the present case suggests that NSCLC harboring uncommon *EGFR* mutations may be a highly heterogenous entity and combined therapeutic strategies such as afatinib plus sequential chemotherapy would be beneficial based on appropriately timed rebiopsies from recurrent lesions.

## Disclosure

The authors have no conflicts of interest to declare.
